# Construction and characterization of a synthetic Baculovirus-inducible 39K promoter

**DOI:** 10.1186/s13036-018-0121-8

**Published:** 2018-12-04

**Authors:** Zhan-Qi Dong, Zhi-Gang Hu, Hai-Qing Li, Ya-Ming Jiang, Ming-Ya Cao, Peng Chen, Cheng Lu, Min-Hui Pan

**Affiliations:** 1 State Key Laboratory of Silkworm Genome Biology, Southwest University, Chongqing, 400716 China; 2 Key Laboratory for Sericulture Functional Genomics and Biotechnology of Agricultural Ministry, Southwest University, Chongqing, 400716 China; 3 Joint National Laboratory for Antibody Drug Engineering, Institute of Immunology, Henan University School of Medicine, Kaifeng, 475004 China

**Keywords:** *Baculovirus*, 39 K, Inducible promoter, Synthetic

## Abstract

**Background:**

Silkworm genetic engineering is widely used in gene function, silk engineering and disease-resistant engineering in most of Asia. Some of the earliest promoter elements are used to control the development of silkworm transgenic expression and gene therapy. However, the low expression and specificity of natural promoters limit the applications of genetic engineering. To construct a highly efficient synthetic inducible promoter in the *Bombyx mori* (Lepidoptera), we analyzed the regulatory elements and functional regions of the *B. mori* nucleopolyhedrovirus 39 K promoter.

**Results:**

Truncated mutation analysis of the 39 K promoter showed that the transcriptional regulatory region spanning positions − 573 to − 274 and + 1 to + 62 are essential for virus-inducible promoter activity. Further investigations using the electrophoretic mobility shift assay revealed that the baculovirus IE-1 protein binds to the 39 K promoter at the − 310 to − 355 region, and transcription activates the expression of 39 K promoter assay. Finally, we successfully constructed a synthetic inducible promoter that increased the virus-inducing activity of other promoters using the baculovirus-inducible transcriptional activation region that binds to specific core elements of 39 K (i.e., spanning the region − 310 to − 355).

**Conclusions:**

In summary, we constructed a novel, synthetic, and highly efficient biological tool, namely, a virus-inducible 39 K promoter, which provides endless possibilities for future research on gene function, gene therapy, and pest control in genetic engineering.

**Electronic supplementary material:**

The online version of this article (10.1186/s13036-018-0121-8) contains supplementary material, which is available to authorized users.

## Background

The inducible promoter, known as the inducible regulation sequence or the inducible enhancer, is a group of promoters that can enhance the expression of exogenous genes under the stimulation of specific physical, chemical, or pathogen signals [1]. In general, the inducible promoter, similar to the transcriptional activator, exists in an inactive form and can be directly or indirectly activated by the corresponding signal. Currently, several technical methods supported by inducible promoters (e.g., Cre-loxp, Tet-On/Tet-Off, and ecdysone and pathogen inducible systems) are widely used in the fields of animal and plant genetic engineering, including gene function identification and a variety of improvements [2–5]. Insects are the largest group of organisms on earth. Some insects such as silkworms and bees are of important economic value. However, a highly efficient inducible system that can be extensively used in insect genetic engineering research has not been established to date, and thus it is of utmost significance to construct a pathogen-inducible promoter in disease resistance breeding and gene therapy [6, 7].

The synthetic promoter provides stronger levels of transcription than natural promoters, as it combines a unique combination of different promoter elements and replace or redesigns sequences with various combinations of promoters [8–10]. Previous studies on synthetic promoters in plants have mainly focused on synthetic inducible promoters [11]. Synthetic promoters have mainly been constructed using cis-regulatory elements that bind to core promoters [12]. The construction of different pathogen-inducible promoters may effectively improve the disease resistance in transgenic plants [11, 13]. Alternatively, constructing an inducible promoter in combination with a tissue-specific promoter (e.g., root, stem, leaf) and inducible promoter contributes to specific tissue-induced expression to improve crop quality, crop robustness, and disease resistance [14]. Synthetic promoters have also been reported in animals [10]. The construction of these synthetic promoters has mainly involved the same direction of assembly of different expression control sequences, application to targeted therapy of diseases, and specific tissue expression of foreign genes [15–17]. Synthetic promoters have recently been studied in insect research, particularly insect disease breeding.

We previously screened the activity of the *B. mori* nucleopolyhedrovirus (BmNPV)-induced promoter (VP1054, P33, Bm21, Bm122, 39 K, P143 and P6.9), and found that the 39 K promoter had the highest BmNPV-induced transcriptional activity [18]. Previous studies have shown that the baculovirus *Autographa californica* nuclearpolyhedrosisvirus (AcMNPV) *39 k* gene is a delayed-early gene that is expressed in infected but not uninfected cells [19]. Mutations in the core region of the 39 K promoter showed that early transcription of AcMNPV 39 K is controlled by two distinct TATA elements and an upstream CAGT sequence as an upstream regulatory region [20]. Further analyses of transcriptional activation revealed that AcMNPV IE0 and IE1 could transactivation expression of the baculovirus 39 K promoter [21]. Previous studies have demonstrated that the AcMNPV 39 K promoter has great utility for insect cell engineering [22]. However, other than in antiviral research, the BmNPV 39 K promoter has not been widely reported.

In our previous study, we found that virus-inducing activity of the BmNPV 39 K promoter could be further increased using enhancers such as Hr3, Hr5, Polh and PU [18]. Simultaneously, overexpression of an exogenous *hycu-ep32* gene controlled by an inducible 39 K promoter showed high antiviral capacity in transgenic lines [23]. Furthermore, we constructed a baculovirus-inducible RNA interference (RNAi) system that inhibits BmNPV replication, is tightly controlled by viral infection, and is not toxic to host cells [24]. Moreover, a highly efficient CRISPR/Cas9 gene editing system was constructed with reduced potential off-target effects and high editing efficiency using the virus-inducible 39 K promoter, which enhanced the antiviral ability of *B. mori* cells [25]. Therefore, to improve the efficiency of the virus-inducible 39 K promoter for gene function studies, silkworm resistance breeding, and pest control, it is imperative to construct a synthetic promoter in insects.

Therefore, in this study, we constructed a synthetic inducible promoter by identifying the 39 K promoter regulatory regions and binding sites. First, we verified the functional domains (spanning regions − 573 to − 274 and + 1 to + 62) of the 39 K promoter by gradually introducing truncating deletions at the 5′ end, 3′ end, and intermediate regions based on characteristics of the 39 K promoter regulatory region as indicated by the dual luciferase report system assay. Then, we constructed a promoter with a shorter promoter sequence and better induction activity by analyzing the regulatory elements of the 39 K promoter and associated point mutations. Furthermore, we found that the baculovirus IE-1 protein binds to the 39 K promoter at the − 310 to − 355 region. Finally, we analyzed the 39 K promoter-inducing active region combined with specific promoters to construct inducible promoters that could efficiently and specifically activate other promoter inducible expression. The results showed that we successfully constructed a synthetic inducible promoter 39 K that could be effectively applied to research on insect gene function, disease resistance breeding, and pest control.

## Results

### Structural and functional analyses of the 39 K promoter

**Fig. 1:**
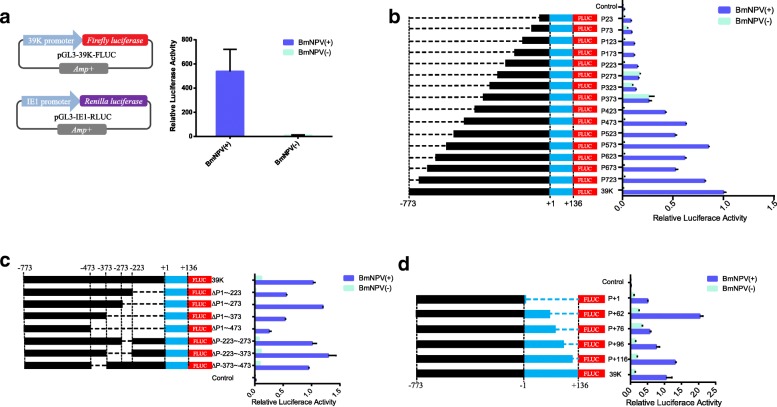
Structural and functional analyses of the 39 K promoter **a** Schematic of *Firefly luciferase* and *Renilla luciferase* expression vectors used for the dual luciferase reporter system. Luciferase activity assay of the dual luciferase reporter system. BmN-SWU1 cells were transfected with pGL3-39 K-FLUC cassettes and the luciferase reporter plasmid pGL3-IE1-RLUC, luciferase activity was measured after infection or non-infection BmNPV. The results were calculated as relative luciferase activity (i.e., *Firefly luciferase*/*Renilla luciferase*). Assays were performed in triplicate. BmNPV(+) represents infection with BmNPV, BmNPV(−) represents non-infection with BmNPV, And NS meansnot significant. Statistically significant differences. **b** Relative luciferase assay of the 5´-end truncated of the 39 K promoter. **c** Relative luciferase assay deletion and truncated fragment of the 39 K promoter. **d** Relative luciferase assay 3´-end truncated of the 39 K promoter. Cells co-transfected with the *Firefly luciferase* and *Renilla luciferase* expression vector were infected or non-infected with BmNPV at 10 MOI. Cells were examined under luciferase reporter system at 48 h p.i.. Black represents − 773~ − 1 fragment, blue represents + 1~ + 136 fragment and dashed line represents the missing fragment of the 39 K promoter. Red represents the *Firefly luciferase* reporter gene. The Y axis represents different truncated promoters and the X axis represents relative promoter activity of different promoters under infectious and non-infected conditions. The results were calculated as the relative luciferase activity (i.e., *Firefly luciferase*/*Renilla luciferase*). The 39 K promoter luciferase activity represents 1 and the promoter activity of the other truncated fragment is a ratio relative to 39 K promoter. BmNPV(+) represents infection with BmNPV, BmNPV(−) represents non-infection with BmNPV

To generate optimized virus-inducible specific promoters, a truncation and mutation strategy was employed to gradually remove the 39 K promoter core region, followed by analysis of changes in 39 K promoter activity. After the 39 K promoter-controlled *Firefly luciferase* and reference plasmid IE1 promoter-controlled *Renilla luciferase* were co-transfected into the BmN-SWU1 cells, *Renilla* luciferase as the amount of protein used for relative luciferase activity. Promoter activity was assessed by detecting changes in *Firefly luciferase* activity relative to that of *Renilla luciferase* (Fig. 1a). Luciferase assay data demonstrated that BmNPV infection efficiently upregulated *Firefly* luciferase activity compared to the non-infection control. Specifically, *Firefly luciferase* activity increased by 64.16-fold in cells infected with BmNPV (Fig. 1a). In contrast, no difference was detected in the non-infected with BmNPV cells. These results show that the luciferase assay system can be applied for the identification and detection of promoter activity. To identify the core areas required for high expression, deletion mutants were created. Using − 773~ + 136 as the original sequence of the 39 K promoter, each truncation was reduced by 50 bp relative to the original sequence (Fig. 1b). Fifteen 5′-truncated luciferase assay plasmids of the 39 K promoter and the reference pGL3-IE1-Rluc plasmid were co-transfected into the BmN-SWU1 cells. At 48 h post-transfection (h p.t.), the luciferase activity was evaluated by adding BmNPV or culture medium, and incubating for 48 h. The results showed a gradual decrease in promoter activity with shorter promoter length. The length of the P573 promoter was shorter by 200 bp relative to the 39 K promoter, but promoter activity only decreased by 14.5% (Fig. 1b). Fragment − 773~ − 573 exhibited little effect on 39 K promoter activity. However, the activity of the P323 promoter decreased by 97.21% relative to the 39 K promoter. These findings suggest that the − 323~ − 573 fragment harbors an important regulatory element of the 39 K inducible promoter. The plasmids P273, P323, and P373 showed strong constitutive promoter activity, and that of the P273 promoter was 12.27-fold higher than P223, indicating that the − 223-273 fragment was related to the constitutive activity of the 39 K promoter (Fig. 1b).

To further analyze the 39 K promoter regulatory motif, an intermediate deletion fragment of the 39 K promoter was created. The ∆P-1~ − 273, ∆P-223~ − 273, ∆P-223~ − 373 and ∆P-373~ − 473 motifs had no significant effects on 39 K promoter activity (Fig. 1c). Further promoter deletion fragments of ∆P-1~ − 223, ∆P-1~ − 373, and ∆P-1~ − 473, led to a rapid decrease in promoter activity (Fig. 1c). Therefore, combined with the 5′-end deletion results and the principle of selecting optimal promoters, the − 1 to − 273 fragment of the 39 K promoter can be deleted to construct of an artificial inducible 39 K promoter. The + 1~ + 136 fragment of the 39 K promoter is the core region, and the 3′ end was gradually truncated and the promoter activity was analyzed. The results showed that the promoter activities of P + 116 and P + 62 increased by 35.4 and 97.00% compared to 39 K, respectively. These results indicated that deletion of + 136~ + 116 and + 76~ + 62 increased the activity of the 39 K promoter (Fig. 1c); thus, these two fragments imparted inhibitory effects on promoter activity. Therefore, the optimal promoter would have the + 136 to + 62 fragments deleted from the 3′ end.

### Construction of an artificial inducible 39 K promoter

**Fig. 2:**
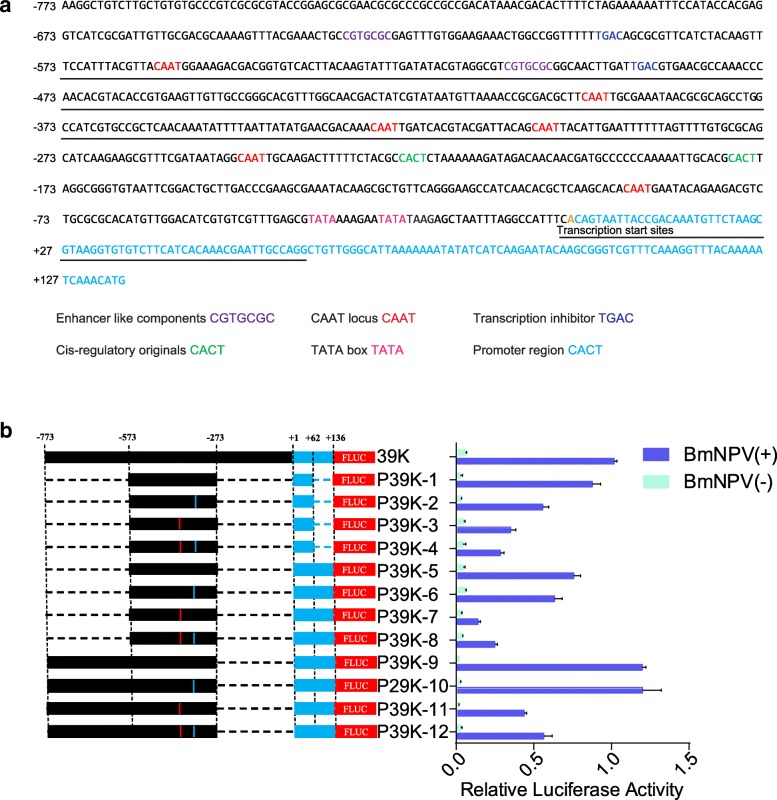
Construction of an artificial inducible 39 K promoter. **a** Analysis of 39 K promoter regulatory element. Purple represents enhancer like components CGTGCGC element, red represents CAAT locus, blue represents transcription inhibitor TGAC box, green represents cis-regulatory original CACT element, and pink represent TATA boxes. Artificial inducible 39 K promoter sequences are underlined. **b** Relative luciferase assay of the artificial inducible 39 K promoter. BmN-SWU1 cells were co-transfected with the indicated *Firefly luciferase* and *Renilla luciferase* expression vector and infected with BmNPV at 10 MOI or uninfected. At 48 h p.i., cells were examined using a luciferase reporter system. Black represents − 773~ − 1 fragment, blue represents + 1~ + 136 fragment and dashed line represents the missing fragment of the 39 K promoter. The regions − 773, − 573, − 273, + 1, + 62 and + 136 represent truncation sites of the corresponding promoter. Red represents the *Firefly luciferase* reporter gene. The red location represents the CAAT mutation to CGGT of 39 K promoter − 399 site. The blue location represents the CAAT mutation to CGGT of 39 K promoter − 329 site. The Y axis represents different truncated promoters and the X axis represent relative promoter activity of different promoters under infectious and uninfected conditions. The results were calculated as the relative luciferase activity (i.e., *Firefly luciferase*/*Renilla luciferase*). The 39 K promoter luciferase activity represents 1 and the promoter activity of the other truncated fragment is a ratio relative to 39 K promoter. BmNPV(+) represents infection with BmNPV, BmNPV(−) represents non-infection with BmNPV. Each data point was determined from the mean of three independent replicates

We performed deletion analysis of the 39 K promoter to identify the regions that effected promoter activity. In addition, we analyzed the key regulatory elements in the core region of the promoter using a promoter prediction program. Online analysis showed that the 39 K promoter contains core components including two enhancer-like components CGTGCGC, six CAAT loci, two transcription inhibitors TGAC, two *cis*-regulatory originals CACT, and two TATA boxes (Fig. 2a). In combination with the position of the 39 K promoter core element and key regulatory regions, we first constructed three artificial inducible promoters, namely P39K-1 (− 573~ − 273 and + 1~ + 62 fragments), P39K-5 (− 573~ − 273 and + 1~ + 136), and P39K-9 (− 773~ − 273 and + 1~ + 136). The activities of the P39K-1, P39K-5 and P39K-9 promoters were 87.24, 75.94, and 112.34% of that of the 39 K promoter, respectively (Fig. 2b). The promoter lengths of the P39K-1, P39K-5, and P39K-9 promoters were 362 bp, 436 bp, and 636 bp, respectively. The purpose of constructing an artificial promoter was to minimize the length of the promoter without affecting its activity. Therefore, the length of the P39K-1 promoter was only 39.91% of the 39 K promoter sequence, but the promoter activity still reached the original 87.24%, which was the better artificially induced promoter. A previous study showed that mutations from CAAT to CGGT significantly increased the promoter activity [26]. To demonstrate that the CAAT motifs of the BmNPV 39 K promoter are involved in transcription activation, we constructed four promoters with mutations at four CAAT motifs in the 39 K promoter. The luciferase results showed that the mutations improved transcriptional activation at the four CAAT motifs. In particular, the mutations of CAAT site to CGGT at the − 326 and − 399 loci increased the activity of the 39 K promoter by 3- and 4-fold, respectively (Additional file 1: Figure S1). Therefore, we constructed nine artificial inducible promoters with − 326 loci, − 399 loci, and two simultaneous mutations in the P39K-1, P39K-5, and P39K-9 promoters, respectively. Dual luciferase reporter assays showed that the promoters with these nine point mutations did not have significantly increased promoter activity relative to the P39K-1, P39K-5 and P39K-9 promoters (Fig. 2b). The P39K-1 artificially inducible promoter still contained enhancers such as component CGTGCGC, the CAAT locus, and the transcription inhibitor TGAC (Additional file 1: Figure S2). These results indicate that P39K-1 still possessed the original promoter regulatory mechanism and thus served as an optimized artificial inducible promoter.

### Identification of inducible promoter 39 K-regulated genes

**Fig. 3:**
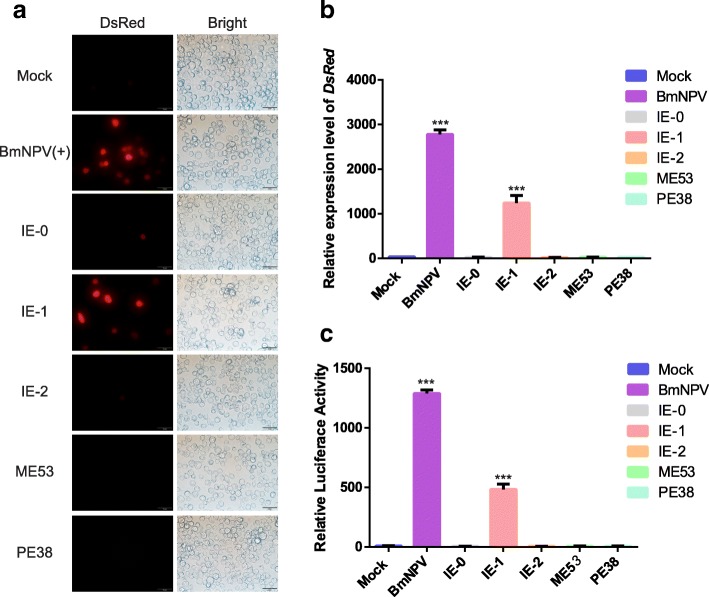
Identification of inducible promoter 39 K-regulated genes. **a** Immunofluorescence analysis of 39 K promoter activated foreign protein expression. p39K-DsRed plasmid co-transfection with immediate early genes and examined under a fluorescence microscope at 96 h p.i. Red represents DsRed protein expression, white represents the number of cells. The letters on the right represent cells of the corresponding early gene overexpressed or BmNPV infection. Mock represents cells not infected with BmNPV or early gene expression. **b** Transcription of inducible p39K-DsRed system with BmNPV immediate early genes. Transient co-expression of p39K-DsRed plasmid and immediate early gene or infected with BmNPV at 10 MOI. At 48 h p.i., total RNA was isolated from each transfected cell and quantified by RT-PCR. Each data point was determined from the mean of three independent replicates. **c** Relative luciferase assay of inducible p39K-DsRed system with BmNPV immediate early genes. Cells co-transfected with the *Firefly luciferase* and *Renilla luciferase* expression vector were infected or non-infected with BmNPV at 10 MOI. Cells were examined at 48 h p.i.. Each data point was determined from the mean of three independent replicates. *** represent statistically significant differences at the level of *P* < 0.001

Expression of the baculovirus gene is regulated by the cascade, and subsequent phase gene expression is dependent on the previous phase [27]. The baculovirus *39 K* gene is a delayed early expression gene [21]. To identify the 39 K promoter transcriptional control gene, we first screened the transcriptional regulation of the 39 K promoter by analyzing five immediate-early genes (i.e., *ie-0*, *ie-1*, *ie-2*, *pe38* and *me53*). We co-transfected pIZ-IE0, pIZ-IE1, pIZ-IE2, pIZ-PE38 and pIZ-ME53 with p39K-DsRed and then detected DsRed at the transcriptional levels. The expression of DsRed protein was only observed in the viral-infected and pIZ-IE1 transfected BmN-SWU1 cells, but not in the pIZ-IE0, pIZ-IE2, pIZ-PE38, pIZ-ME53, and non-infected cells (Fig. 3a). These results indicate that the DsRed protein is rapidly activated by viral infection and IE-1 protein expression. Moreover, to detect the sensitivity of the inducible promoter, we evaluated the transcription of DsRed induced by viral protein and BmNPV. The results showed that the virus and IE-1 protein induced large-scale transcription of *dsred* (Fig. 3b). However, no changes in *dsred* transcription levels in the pIZ-IE0, pIZ-IE2, pIZ-PE38, and pIZ-ME53-transfected and non-infected cells were observed. The luciferase assay also showed that only the IE-1 protein could effectively induce 39 K promoter activity (Fig. 3c). In addition, the other early genes were not transcriptionally regulated by the 39 K promoter.

### Analysis of IE-1 binding to the 39 K promoter region

**Fig. 4:**
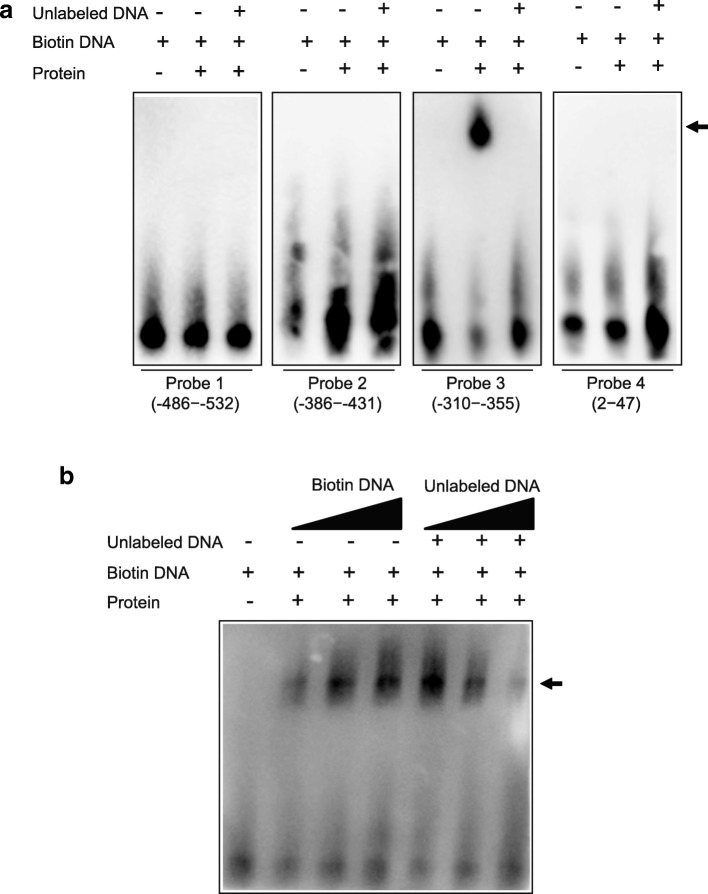
EMSA analysis of IE-1 binding to 39 K promoter region. **a** Electrophoretic mobility shift assay indicated that the 39 K probes bind to the recombinant IE-1 proteins. We used the competitive inhibitors unlabeled DNA probes as control and without IE-1 proteins as negative control. The shift of the positive control is indicated by a thick stripe. We detected probes 3 (− 310~ − 355) with block stripes. On the contrary, probe 1 (− 486~ − 532), probe 2 (− 386~ − 431), and probe 4 (+ 2~ + 47) were not associated with IE-1. **b** The EMAS detected that the probe 3 (− 310~ − 355) binds to the recombinant IE-1 proteins. The probe 3 (− 310~ − 355) probes concentrations were 1, 2, and 6 pmol/L; the IE-1 protein concentration was 0.8 μg/L; the concentrations of compete probes were 2, 20, and 100 pmol/L.

To further strengthen the case that IE-1 is a direct transcriptional target of the 39 K promoter, we performed a gel-shift competition assay using a biotin-labeled oligonucleotide spanning the potential IE1-binding sequence as probe. Through online program prediction, we designed a total of four probes containing multiple potential binding sites, namely probe 1 (− 486~ − 532), probe 2 (− 386~ − 431), probe 3 (− 310~ − 355), and probe 4 (+ 2~ + 47), which were incubated with purified IE-1 derived from prokaryotic expression. The incubation of the biotin with the IE-1 protein. The incubation of biotin labeled probe 3 (− 310~ − 355) with IE-1 protein resulted in a distinct band shift in the electrophoretic mobility shift assay (EMSA), which disappeared with the addition of competitive unlabeled DNA probes (Fig. 4a). In contrast, no significant band shift was detected in the EMSA after incubation with probe 1 (− 486~ − 532), probe 2 (− 386~ − 431) and probe 4 (+ 2~ + 47) (Fig. 4a).

To further examine the binding activity of probe 3 with the IE-1 proteins, we analyzed the effect of the biotin labeled probe and unlabeled DNA on band shifting. The incubation of probe 3 with IE-1 protein resulted in a band shift, which increased with increasing concentrations of biotin labeled probe 3 and decreased with increasing concentrations of competitive unlabeled DNA probe (Fig. 4b). No significant band shift was detected in the probe without incubation with the IE-1 proteins. There results indicated that IE-1 specifically bound to 39 K promoter probe 3 (− 310 to − 355) during transcriptional activation of the BmNPV IE-1 protein-inducible 39 K promoter.

### Application of the artificial inducible 39 K promoter

Transgenic overexpression of exogenous genes and gene editing are important methods for improving disease resistance in the silkworm. To determine if the synthetic inducible promoter can be used in transgenic breeding, we attempted to use synthetic baculovirus inducible 39 K promoter for exogenous gene overexpression and gene editing system. After transfecting pIZ-P39K-1-DsRed and pIZ-P39K-1-Cas9 into BmN-SWU1 cells, the transcription and expression levels of the cas9 gene were detected by qPCR, respectively. The results showed the robust expression of cas9 gene transcripts from 12 h post-infection (h p.i.) (Additional file 1: Figures S3a and b).

**Fig. 5:**
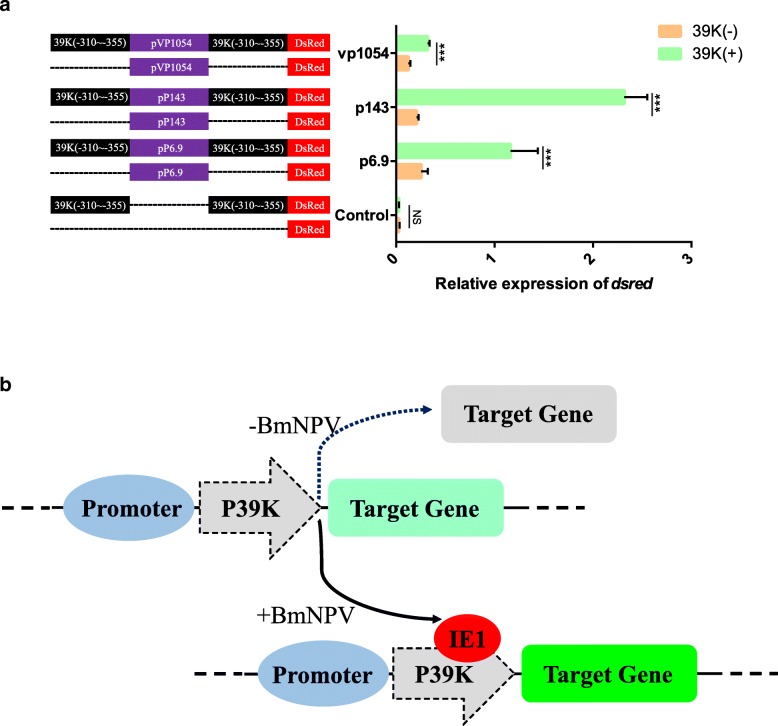
Application of the artificial inducible 39 K promoter. **a** qPCR assay of the artificial inducible 39 K (− 310~ − 355) transcriptional activation region increased promoter activity. BmN-SWU1 cells were co-transfected with indicated expression vector and infected with BmNPV at 10 MOI or uninfected. At 48 h p.i., cells were examined. Black represents 39 K(− 310~ − 355) fragment, purple represents the corresponding promoter sequence, red represents the *dsred* gene, dashed line represents the missing fragment of the 39 K promoter. The Y axis represents different artificial inducible promoters and the X axis represents relative promoter activity of different promoters under infected conditions. Each data point was determined from the mean of three independent replicates. NS, not significant. ** represent statistically significant differences at the level of *P* < 0.001. **b** Schematic plot of the synthetic inducible promoter

To expand the potential use of artificial-inducible 39 K promoters in insect genetic engineering, we synthesized new promoters by combining other baculovirus promoters and 39 K(− 310~ − 355) binding sequences. qPCR assays indicated that the baculovirus VP1054, P143 and P6.9 promoters exhibited a significant increase in activity compared to the original sequence after binding to the 39 K(− 310~ − 355) sequence (Fig. 5a). The promoter activity of VP1054, P143 and P6.9 increased by 2.55-, 11.15-, and 4.64-fold than the original sequence (Fig. 5a). These results demonstrate that the 39 K promoter fragment can be utilized in the construction of an artificially inducible promoter to increase induction activity in genetic engineering.

## Discussion

Naturally occurring promoters are currently used in protein production and gene therapy [28, 29]. However, they are not always capable of driving high levels of gene expression and may also lack the required specificity depending on the promoter and the specific application [28, 30]. As genetic engineering goals become more elaborate and targeted, more precise gene expression tools will be needed [31, 32]. Synthetic promoters contain fragments of natural promoters to form new DNA sequence fragments that are not found in nature and are more powerful and specific than naturally occurring promoters [10, 32]. Considering that scientists have been engineering silkworms for more than 20 years and that silkworm genetic engineering has been widely used in gene function, silk engineering, and disease resistance breeding in most of Asia, it is surprising that we are still using some of the earliest-developed tools to control transgene expression in silkworms [33–36]. To more effectively and specifically apply silkworm genetic engineering, we constructed a highly efficient synthetic baculovirus-inducible 39 K promoter. The 39 K (− 310~ − 355) sequence is widely used to enhance promoter activities to construct synthetic inducible promoters provides an efficient tool for synthetic biology and genetic engineering.

In our previous studies, we showed that the P-44 (− 44 to + 133) and P-420 (from − 420 to − 611) are important regions for the transcriptional activation of the 39 K promoter, although the activity of the 39 K promoter induced by the transcriptional regulatory region was not studied in detail [18]. To obtain a synthetically inducible promoter with a shorter sequence and better induction activity, we performed stepwise analysis of the 39 K promoter transcriptional regulatory region to identify the influence of different regions on 39 K promoter and induction activity. In combination with the above promoter activity analysis, we constructed three artificial inducible promoters P39K-1 (− 573~ − 273 and + 1~ + 62 fragments), P39K-5 (− 573~ − 273 and + 1~ + 136), and P39K-9 (− 773~ − 273 and + 1~ + 136). Previous studies have shown that mutation of the *Autographa californica* multiple nucleopolyhedrovirus (AcMNPV) ubiquitin promoter CAAT to CGGT increases inducible promoter activity [26]. In this study, we compared mutations in the CAAT site of each artificial inducible promoter, and found that they did not lead to a significant increase compared to the original promoter (Fig. 2). Therefore, an optimal synthetic inducible promoter for P39K-1 was constructed in this study. The promoter length of P39K-1 was only 33% of the P39K promoter, without a significant decrease in promoter activity relative to the P39K promoter. The reduction of such long promoter fragments provides significant improvement to the field of genetic engineering.

The AcMNPV 39 K promoter is mainly expressed by immediate early genes [37–39]. To systematically analyze transcriptional activation of BmNPV, we analyzed the expression of the 39 K promoter as induced by different early transcriptional activator factors. The results showed that only the IE-1 could induce 39 K promoter initiation activity, but that of IE-0 and IE-2 did not, unlike the AcMNPV 39 K promoter (Fig. 3) [21, 38, 39]. The promoter-specific application could be significantly improved by the expression of the promoter according to the binding specificity of the inducible promoter regulatory sequence in plant genetic engineering and mammalian gene therapy applications [5, 17, 32]. Here, we demonstrated that the 39 K (− 310~ − 355) sequence can be applied to the construction of artificial inducible promoters (Fig. 5b). Furthermore, we could also use the original sequence to increase the induction activity of other weakly expressed promoters or increase the inducible activity of a promoter by repeating this fragment several times. Meanwhile, the combination of different promoter regulatory elements could also be used to improve the activity of synthetic inducible promoters. In our previous studies, we successfully applied the virus-inducible promoter 39 K to transgenic overexpressing foreign genes, RNAi, and gene editing, and the determination of this promoter binding region may be more accurately applied to the regulation of genetic engineering [23–25]. These synthetic inducible promoters also allow more extensive applications to biopharmaceutical and agricultural processes and novel gene therapies. The successful construction of baculovirus synthetic inducible promoters provides a new strategy for the research and application of insect genetic engineering, pest control, baculovirus expression systems, and insect bioreactors. In future research, we plan to use the following strategies to improve the scope of applications of virus-inducible promoters: 1) Incorporating inducible promoter regulatory sequences and tissue-specific promoters to synthesize new promoters to induce expression of specific proteins in specific tissues to avoid loss of host energy and cell cytotoxicity. 2) Combination with the 39 K promoter and IE1 protein binding sequence, a foreign protein inducible expression system will be constructed and applied to insect gene function research; and 3) a broad-spectrum pathogen induction system will be constructed to cultivate genetic engineering varieties that may respond to different pathogens. In addition, the optimization of synthesized promoters can further increase the specificity and yield of foreign proteins expressed by baculovirus expression systems, as well as the application of insect pest control, such as the pathogen-inducible transgenic cotton bollworm, *Spodoptera exigua*, and the other economic crop pests.

## Conclusions

In conclusion, we constructed and optimized the synthetic baculovirus-inducible 39 K promoter that could be effectively applied for CRISPR/Cas9 gene editing and transgenic technology to construct transgene material of silkworm and provides an efficient tool for synthetic biology and gene therapy. The synthesized inducible promoters also provide new insights to improve strategies for insect genetic engineering, pest control and gene function research.

## Methods

### Cells and viruses

The *B. mori* ovary cell line BmN-SWU1 was cultured at 27 °C in TC-100 medium (United States Biological, Salem, MA, USA) supplemented with 10% (*V*/V) fetal bovine serum (Gibco, Gaithersburg, MD, USA) and 10% (V/V) penicillin/streptomycin [40]. Recombinant BmNPV (vA4^prm^-EGFP) containing an EGFP marker gene driven by the *B. mori* actin A4 promoter was created from the bacmid bMON7214, which contains the BmNPV genome [41, 42]. The BmN-SWU1 cells were transfected with the vA4^prm^-EGFP construct, and viral titers were determined using the 50% tissue culture infective doses assay [42].

### Plasmid construction

Previous studies have shown that the − 773 bp upstream and + 134 bp downstream motifs of the 39 K promoter transcription initiation site are critical regions for 39 K promoter activity [18]. To analyze the structural features of the 39 K promoter, we performed a stepwise truncation analysis of the 39 K promoter. Truncated fragments of 39 K promoters were cloned into the pGL3-basic vector (Promega, Gaithersburg, MD, USA) to construct the *Firefly luciferase* (FLUC) expression vector. The 5′ truncated plasmids fragment included P-723 (− 773~ − 724 deletion), P-673 (− 773~ − 674 deletion), P-623 (− 773~ − 624 deletion), P-573 (− 773~ − 574 deletion), P-523 (− 773~ − 524 deletion), P-473 (− 773~ − 474 deletion), P-423 (− 773~ − 424 deletion), P-373 (− 773~ − 374 deletion), P-323 (− 773~ − 324 deletion), P-273 (− 773~ − 274 deletion), P-223 (− 773~ − 224 deletion), P-173 (− 773~ − 174 deletion), P-123 (− 773~ − 124 deletion), P-73 (− 773~ − 74 deletion), and P-23 (− 773~ − 24 deletion) (Fig. 1b). The 3′ truncated fragment plasmids included P + 116 (+ 117~ + 136 deletion), P + 96 (+ 97~ + 136 deletion), P + 76 (+ 77~ + 136 deletion), P + 62 (+ 63~ + 136 deletion), and P + 1 (+ 2~ + 136 deletion) (Fig. 1d). The intermediate segment deletion plasmids included ∆P-1~ − 223 (− 1~ − 223 deletion), ∆P-1~ − 273 (− 1~ − 273 deletion), ∆P-1~ − 373 (− 1~ − 373 deletion), ∆P-1~ − 473 (− 1~ − 473 deletion), ∆P-223~ − 273 (− 223~ − 273 deletion), ∆P-223~ − 373 (− 223~ − 373 deletion), and ∆P-373~ − 473 (− 373~ − 473 deletion) (Fig. 1c). Then, the sequence of the IE1 promoter and the *Renilla luciferase* (RLUC) reporter gene were linked to the pGL3 vector, named pGL3-IE1-Rluc and used as internal reference plasmid.

The baculovirus immediate early genes *ie-0*, *ie-1*, *ie2*, *pe38* and *me53* from the BmNPV genome were cloned into a pIZ/V5-His (Invitrogen, Carlsbad, CA, USA) vector to generate pIZ-IE0, pIZ-IE1, pIZ-IE2, pIZ-PE38 and pIZ-ME53. The baculovirus-inducible promoter 39 K was cloned into the pIZ-DsRed to replace the OpIE2 promoter. The resulting p39K-DsRed plasmid was used as vector backbone for baculovirus-inducible expression of DsRed. All clones were verified by sequencing. All primers used in this study are presented in Additional file 1: Table S1.

### Dual luciferase reporter assays

The dual luciferase expression plasmids pGL3-39 K-Fluc (450 ng) and pGL3-IE1-Rluc (50 ng) were co-transfected into the BmN-SWU1 cells. Approximately 24 h later, these were infected with BmNPV at multiplicity of infection (MOI) =10. At 72 h p.i., the cells were collected, and luciferase activities were measured with using Dual-Glo Luciferase Assay Kit (Promega) using the ultra-high sensitivity fluorescence chemiluminescence detector. Relative luciferase activity (FLUC/RLUC) was normalized to values obtained using pGL3-39 K-Fluc as control plasmid. Each experiment analysis was repeated three times.

### Transfection and fluorescence analysis

The BmN-SWU1 cells were cultured in 24-well plates (Corning, Corning, NY, USA). After the cells had stabilized, BmNPV immediate-early gene expression plasmids pIZ-IE0, pIZ-IE1, pIZ-IE2, pIZ-PE38 and pIZ-ME53 (0.4 μg) with the p39K-DsRed (0.4 μg) plasmid were co-transfected into cells using the X-tremeGENE HP DNA Transfection Reagent (Roche, Switzerland). At 48 h p.t., all cells were visualized on an Olympus inverted fluorescence microscope with the same parameter settings.

### qPCR

After the BmNPV immediate early gene expression plasmid pIZ-IE0, pIZ-IE1, pIZ-IE2, pIZ-PE38 or pIZ-ME53 with the p39K-DsRed plasmid were co-transfected into the cells, total RNA was isolated using the TRIzol RNA Extraction Kit (Thermo Fisher Scientific, Waltham, MA, USA), following the manufacturer’s instructions. RT-PCR were performed with an iTaqTM Universal SYBR® Green Supermix and CFX Connect Real-Time PCR Detection System (Bio-Rad, Hercules, CA, USA) using primers specific for DsRed (Additional file 1: Table S1). The *Bombyx mori sw22934* gene was used as the reference. The reaction conditions of the RT-PCR reactions were as follows: 95 °C for 30 s; followed by 40 cycles at 95 °C for 5 s and 60 °C for 20 s with 1 M of each primer. All experiments were repeated three times.

### Recombinant expression and protein purification

The coding region of IE-1 was amplified with specific primers IE1-F/IE1-R and cloned into the pCold-I vector and the pGX-4 T-1 vector. Positive plasmids were transformed into *E. coli* strain BL21 competent cells and induced with 0.3 mM, 0.5 mM and 1.0 mM, of IPTG to express the IE1-His recombinant protein. The IE1-His protein was purified using a His-Trap HP column (GE Healthcare, Freiburg, Germany), according to the manufacturer’s recommendations.

### Analysis by EMSA

To analyze the potential binding sites of the 39 K promoter, two different transcription factor binding site prediction programs, namely, Neural Network Promoter Prediction (http://www.fruitfly.org/seq_tools/nnppHelp.html) and JASPAR CORE (http://jaspar.genereg.net/) were used. A total of four potential transcription factor binding sites were identified, which were located at positions − 486 to − 532,-386 to − 431,-310 to − 355 and + 2 to + 47 of the 39 K promoter. For EMSA, the probes were 5´-labeled with biotin (Thermo Fisher Scientific), and then the labeled oligonucleotides were annealed to produce a double-stranded probe. All probes used in this study are presented in Additional file 1: Table S2.

To evaluate the interactions between IE-1 proteins and 39 K regulatory elements, EMSA was conducted according to the guidelines of the Light Shift Chemiluminescent EMSA Kit (Thermo Fisher Scientific). After a 30 min incubation at 25 °C, reaction mixtures were loaded onto 6% (*w*/*v*) native polyacrylamide gels and resolved by electrophoresis electrophoresed in TBE buffer (89 mM Tris, 89 mM boric acid, 2 mM EDTA, pH 8.3) for approximately 1 h at 100 V on ice. The proteins were transferred onto a PVDF membrane (Roche). Bound HRP-conjugated bands were visualized using the LightShift Chemiluminescent EMSA Kit according to the manufacturer’s protocol.

### Construction of the artificial inducible 39 k promoter

Based on the results of 39 K promoter truncation analysis, three synthetic inducible promoters were constructed, namely, p39K-1 (contains the + 1~ + 62 and − 273~ − 573 fragments), p39K-5 (contains the + 1 ~ + 136 and − 273~ − 573 fragments) and p39K-9(contains the + 1~ + 136 and − 273~ − 773 fragments). To improve the promoter activity of 39 K, point mutations of the CAAT box to CGGT at position of − 329, − 399, or − 329 and − 399 were created. A total of 12 synthetic inducible promoters were constructed in combination with truncated and point mutation vectors and designated as p39K-1 to p39K-12, respectively. All artificially inducible promoters were synthesized by Genscript (Nanjing, China) and cloned into the pGL3-basic vector.

### Statistical analysis

All data are expressed as the mean ± standard deviation (SD) of three independent biological experiments. Statistical analyses were performed with the Student’s *t* tests using GraphPad Prism6. *P* values less than 0.01 were considered statistically significant.

## Additional files

Additional file 1: **Figure S1.** Relative luciferase assay of the BmNPV 39 K promoter CAAT motifs are involved in transcription activation **Figure S2.** Analysis of P39K-1 promoter regulatory element **Figure S3.** Application of the artificial inducible 39 K promoter to genetic engineering. **Table S1.** Sequences of primers used in this study. **Table S2.** Sequences of probes used in this study. (DOCX 156 kb)

## Abbreviations

AcMNPV: *Autographa californica* multiple nucleopolyhedrovirus
BmNPV: *Bombyx mori* nucleopolyhedrovirus
CRISPR: Clustered regularly interspaced short palindromic repeats
DsRed: *Discosoma sp.* red fluorescent protein
FLUC: Firefly luciferase
RLUC: *Renilla luciferase*
RT-qPCR: Reverse Transcription- quantitative Polymerase Chain Reaction
TCID_50_: 50% tissue culture infective doses
