# Supplementary material for: Construction and characterization of a synthetic Baculovirus-inducible 39K promoter
Source: J Biol Eng. 2018 Dec 4;12:30. doi: 10.1186/s13036-018-0121-8 (PMC6280533; doi:10.1186/s13036-018-0121-8)
Supplement: Supplementary file 1 — Figure S1. Relative luciferase assay of the BmNPV 39 K promoter CAAT motifs are involved in transcription activation Figure S2. Analysis of P39K-1 promoter regulatory element Figure S3. Application of the artificial inducible 39 K promoter to genetic engineering. Table S1. Sequences of primers used in this study. Table S2. Sequences of probes used in this study. (DOCX 156 kb) [file 13036_2018_121_MOESM1_ESM.docx]

**Construction and Characterization of a Synthetic Baculovirus-inducible 39K Promoter**

Zhan-Qi Dong^a^†, Zhi-Gang Hu^a^†, Hai-Qing Li^a^, Ya-Ming Jiang^a^, Ming-Ya Cao^c^, Peng Chen^a^, Cheng Lu^a,b#^, Min-Hui Pan^a,b#^

State Key Laboratory of Silkworm Genome Biology, Southwest University, Chongqing 400716, China^a^;

Key Laboratory for Sericulture Functional Genomics and Biotechnology of Agricultural Ministry, Southwest University, Chongqing 400716, China^b^;

Joint National Laboratory for Antibody Drug Engineering, Institute of Immunology, Henan University School of Medicine, Kaifeng 475004, China.^c^;

Running head: Construction of a Synthetic Inducible 39K Promoter

†These authors contributed equally to this work.

^#^Address correspondence to Cheng Lu, lucheng@swu.edu.cn, and Min-Hui Pan, pmh047@ 126.com.

**
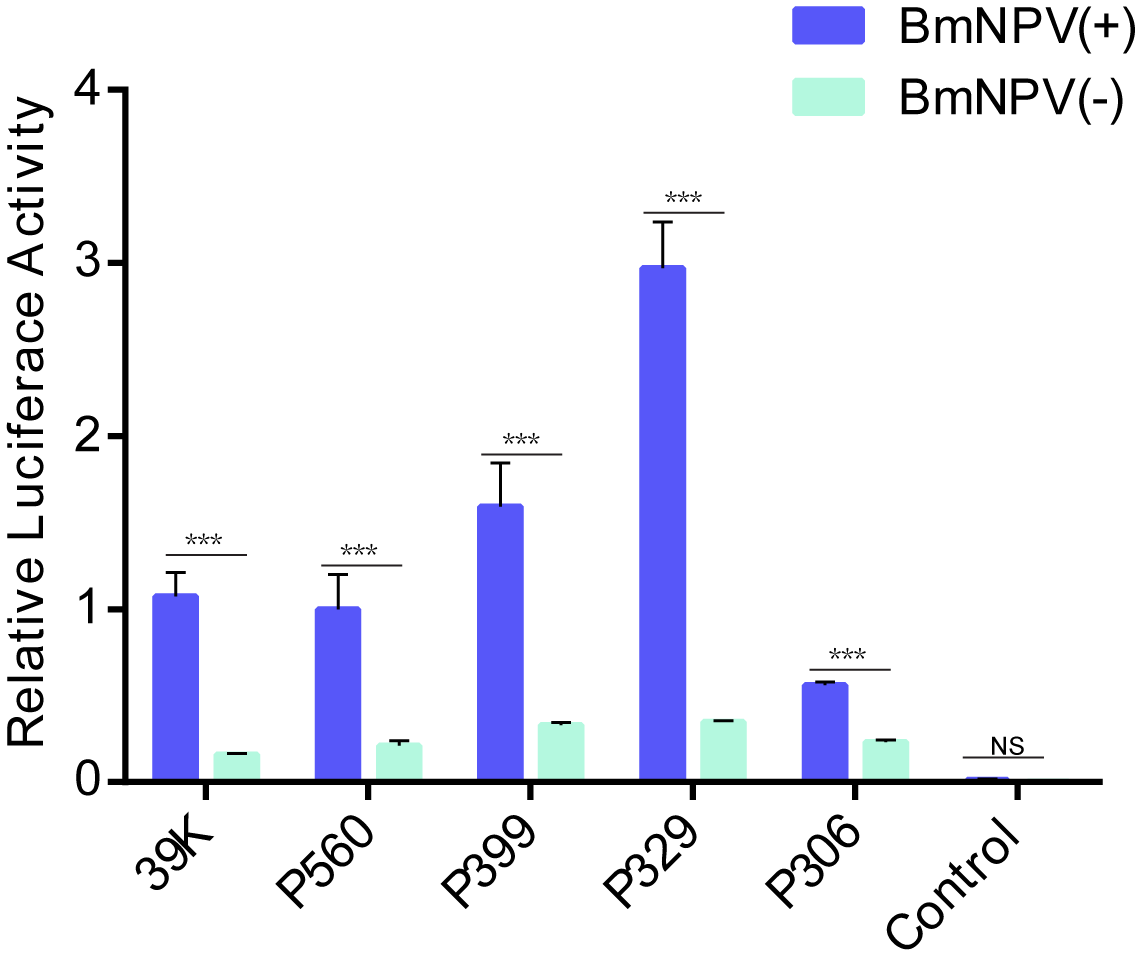
Fig. S1. Relative luciferase assay of the BmNPV 39K promoter CAAT motifs are involved in transcription activation.** BmN-SWU1 cells were co-transfected with indicated *Firefly* luciferase and *Renilla* luciferase expression vector and infected with BmNPV at 10 MOI or uninfected. At 48 h p.i., cells were examined under luciferase reporter system. The X axis represents different mutations of 39K promoters CAAT motifs and the Y axis represents relative promoter activity of different promoters under infected and non-infected conditions. The results were calculated as the relative luciferase activity (i.e., Firefly luciferase/Renilla luciferase). BmNPV(+) represents infection with BmNPV and BmNPV(-) represents non-infection with BmNPV. Each data point was determined from the mean of three independent replicates. NS, not significant. ** represent statistically significant differences at the level of *P*< 0.01.


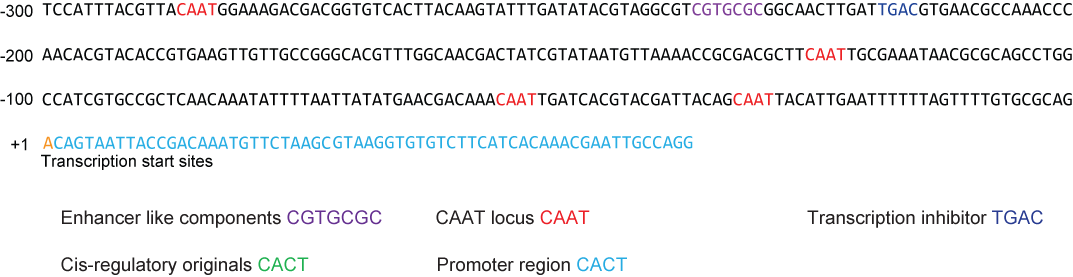


**Fig. S2. Analysis of P39K-1 promoter regulatory element.** Purple represents enhancer like components CGTGCGC element, red represents CAAT locus, blue represents transcription inhibitor TGAC box, green represents cis-regulatory original CACT element.


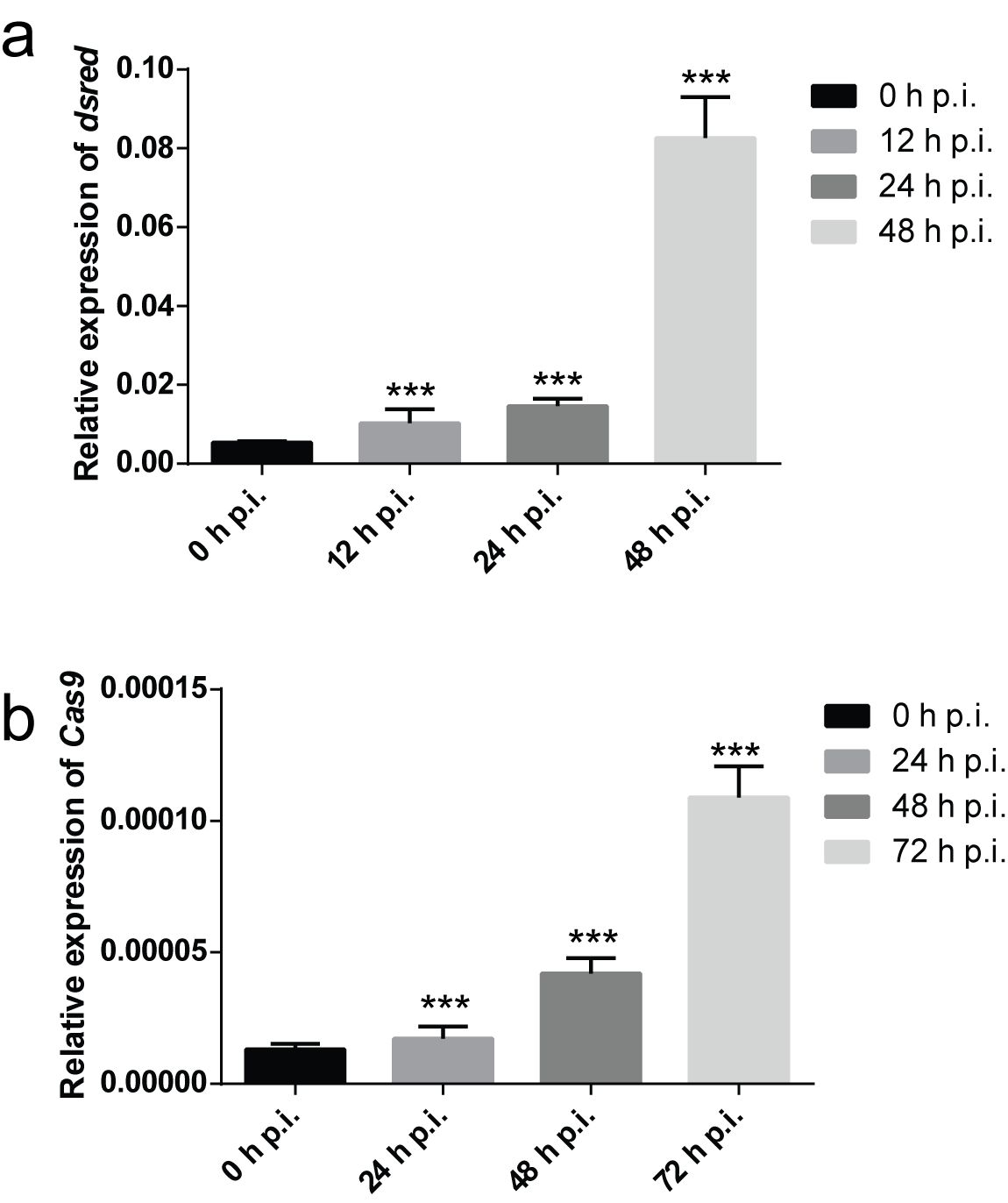


**Fig. S3. Application of the artificial inducible 39K promoter to genetic engineering.** (a-b) Transcription of the synthetic 39K promoter after exogenous gene and CRISPR/Cas9 system infection with BmNPV. BmN-SWU1 cells were transfected with indicated pIZ-P39K-1-DsRed, pIZ-P39K-1-Cas9 and infected with BmNPV at 10 MOI. At 48 h p.i., total RNA was isolated from each transfected group and quantified by qPCR. Each data point was determined from the mean of three independent replicates. NS, not significant. ***represents statistically significant differences at *P* < 0.001.

**Table S1. Sequences of primers used in this study.**

| Primer name | Primer sequence^*^(5'−3') |
| --- | --- |
| Promoter primers | |
| 39K-773(Bgl II)-F | GGAAGATCTAAGGCTGTCTTGCTGTGTGC |
| 39K-723(Bgl II)-F | GGAAGATCTCCGCCGCCGACATAAACG |
| 39K-673(Bgl II)-F | GGAAGATCTGGTCATCGTGATTGTTGCGACGCAA |
| 39K-623(Bgl II)-F | GGAAGATCTGTTTGTGGAAGAAACTGGCCGGT |
| 39K-573(Bgl II)-F | GGAAGATCTTTCCATTTACGTTACAATGGAA |
| 39K-523(Bgl II)-F | GGAAGATCTATATACGTAGGCGTCGTG |
| 39K-473(Bgl II)-F | GGAAGATCTCAACACGTACACCGTGAAGTTG |
| 39K-423(Bgl II)-F | GGAAGATCTGTATAATGTTAAAACCGCGA |
| 39K-373(Bgl II)-F | GGAAGATCTGCCATCGTGCCGCTCAACAAA |
| 39K-323(Bgl II)-F | GGAAGATCTGATCACGTACGATTACAGCAA |
| 39K-273(Bgl II)-F | GGAAGATCTGCATCCAGAAGCGTTTCGATAATAG |
| 39K-223(Bgl II)-F | GGAAGATCTACTCTAAAAAAGATAGACAACAACG |
| 39K-173(Bgl II)-F | GGAAGATCTTAGGCGGGTGTAATTCGGACTGCTT |
| 39K-123(Bgl II)-F | GGAAGATCTTTCAGGGAAGCCATCAACACGCT |
| 39K-73(Bgl II)-F | GGAAGATCTCTGCGCGCACATGTTGGACATCG |
| 39K-23(Bgl II)-F | GGAAGATCTTAAGAGCTAATTTAGGCCATTTCAC |
| 39K~+136(Hind III)-F | CCCAAGCTTCCATGTTTGATTTTTGTAAACCT |
| 39K~+116(Hind III)-R | CCCAAGCTTCTTTGAAACGACCCGCTTGCATTCT |
| 39K~+96(Hind III)-R | CCCAAGCTTATATATTTTTTTAATGCC |
| 39K~+76(Hind III)-R | CCCAAGCTTCTTGATGATATATTTTTTTAATGCC |
| 39K~+62(Hind III)-R | CCCAAGCTTCCTGGCAATTCGTTTGTGATGAAGA |
| 39K~+1-(Hind III)-R | CCCAAGCTTTGAAATGGCCTAAATTAGCTCTTAT |
| RT-PCR primers | |
| RT-DsRed/F | CGAGACGGCTGCACAAAA |
| RT-DsRed/R | TGCCCAAAAGAAACCCACA |
| RT-sw22934/F | TTCGTACTGCTCTTCTCGT |
| RT-sw22934/R | CAAAGTTGATAGCAATTCCCT |
| Overexpression primers | |
| IE0(EcoR I)-F | CGGAATTCATGATAAGAACCAGCAGTC |
| IE0(Not I)-R | ATAAGAATGCGGCCGCTTTATACGATGTCCTGCA |
| IE1(EcoR I)-F | CGGAATTCATGACGCAAATTAATT |
| IE-1(Not I)-R | ATAAGAATGCGGCCGCATTAAATTCAATTTTTTTATAT |
| IE2^HA^(BamH I)-F | CGCGGATCCATGTACCCATACGACGTCCCAGACTACGCTAGTCGCCAAATCAACGC |
| IE2^HA^ (Not I)-R | ATAAGAATGCGGCCGCTTAGGCGTAGTCGGGCACGTCGTAGGGGTA AGGTTTAGACATCTCAATAGTG |
| PE38(EcoR I)-F | CGGAATTCATGGACAAACGTGCC |
| PE38(Not I)-R | ATAAGAATGCGGCCGCCAATTTATCACATTTACG |
| ME53(EcoR I)-F | CGGAATTCATGAACCGTTTTTTT |
| ME53(Not I)-R | ATAAGAATGCGGCCGCGACATTGTTGTTTAC |
| DsRed (HindIII)-F | CCCAAGCTTATGGCCTCCTCCGAGAACG |
| DsRed (XbaI)-R | GCTCTAGACTACAGGAACAGGTGGTGGC |

^*^ (The restriction enzyme sites are marked in red).

**Table S2. Sequences of probes used in this study.**

| Probes name | Probes sequence^*^(5'−3') |
| --- | --- |
| Probes 1 (-486~-532) | AGTATTTGATATACGTAGGCGTCGTGCGCGGCAACTTGATTGACGTG |
| Probes 2 (-386~-431) | GACTATCGTATAATATTAAAACCGCGACGCTTCAATTGCGAAATAA |
| Probes 3 (-310~-355) | AATATTTTAATTATATGAACGACAAACAATTGATCACGTACGATTA |
| Probes 4 (+2~+47) | AGTAATTACCGACAAATGTTCTAAGCGTAAGGTGTGTCTTCATCAC |
